# Assessment of Myometrial Concentrations of Oestrogen and Progesterone Receptors in the Lower Uterine Segment of Full-Term Pregnancies in Presence or Absence of Labour

**DOI:** 10.1155/2013/213193

**Published:** 2013-06-02

**Authors:** Joana Soares de Arruda, Edward Araujo Júnior, Manuel de Jesus Simões, Luiz Kulay Júnior

**Affiliations:** ^1^Department of Obstetrics, Federal University of São Paulo (UNIFESP), Rua Carlos Weber, 956 Apartment 113 Visage, Vila Leopoldina, 05303-000 São Paulo, SP, Brazil; ^2^Department of Morphology, Federal University of São Paulo (UNIFESP), São Paulo, SP, Brazil

## Abstract

*Objective*. To assess the concentration of progesterone (PRs) and oestrogen (ORs) receptors of myometrium of full-term pregnant women in the myometrium of lower segment of the uterus in relationship with presence or absence of labour. *Methods*. This was a cross-sectional prospective study with 21 pregnant women, being 6 in labour (Group I) and 15 without labour (Group II). The biopsy of myometrium was realized during caesarian section, and the excised tissue was stained using immunohistochemical techniques for the quantification of the receptors, and with the aid of image-analysis software, the numbers of receptors for each hormone were determined spectrophotometrically. The Mann-Whitney test was used to compare the pregnant women in each study group with respect to the numbers of ORs and PRs. The Wilcoxon test was used to compare the concentration of ORs and PRs in each group separately. *Results*. The mean of gestational age was 39 weeks, (range, 37 to 41 weeks). The medians of PRs and ORs in pregnant women in labour (Group I) were 29.3 (range, 24.6–30.2) and 32.3 (range, 22.9–49.0), respectively. The medians of PRs and ORs in pregnant women without labour (Group II) were 43.6 (range, 23.6–70) and 43.9 (range, 18.3–62.6), respectively. We did not observe significant differences of the number of ORs and PRs in both groups (*P* = 0.13 and 0.37, resp.). The number of ORs was statistically more than that of PRs in Group II (*Z* calculated = 16.00). *Conclusion*. The concentrations of PRs and ORs were similar in the myometrium of the lower uterine segment of pregnant women during and without labour, but the concentration of ORs was more than that of PRs in the myometrium of the lower uterine segment of pregnant women without labour.

## 1. Introduction

Hormonal changes at the moment of parturition may constitute an important regulatory mechanism for myometrial contraction. In some species (e.g., sheep), sudden changes in circulating cortisol, oestrogen, and progesterone appear to be important factors for the onset of labour, whereas in humans, these signs remain unknown [1–10]. Therefore, we investigated the concentration of oestrogen receptors (ORs) and progesterone receptors (PRs) in pregnant human women because local modifications of these receptors may be associated with the myometrial conditions responsible for the mechanism of parturition [1–10]. 

Prior studies on hormone concentrations during labour have failed to demonstrate significant changes indicating their direct involvement in labour; however, local changes in hormone receptors are believed to be responsible for parturition [4, 5, 11]. Close to term, the effect of progesterone may be inhibited by a genetic mechanism involving one or more agents not associated with receptors [1, 2]. This genetic mechanism may lead to the attenuation of progesterone's effects by, for example, a specific gene that interrupts its action at a molecular, biochemical, and/or cellular level [1]. Corroborating these studies in a review, Mendelson postulates that an increase in inflammatory response occurs as a result of the activation of the nuclear factor (kappa) beta, leading to uterine contractility in two ways: (1) the direct activation of contractile genes (e.g., COX-2, oxytocin receptors, and connexin 43) and (2) a blockade of the ability of progesterone receptors (PRs) to maintain uterine quiescence [5].

According to previous reports, a decrease in the concentration of PRs is observed in patients during labour [4–12]. Nonetheless, the inhibition of the effects of gestagenic agents does not result in the immediate onset of labour, although this inhibition sensitises the cells of the myometrium to agents that induce uterine contractility. In contrast, oestrogen enables the myometrium to prepare for parturition by inducing oxytocin receptors, which is perhaps the first step of parturition [5–10, 12].

Recent studies have also suggested that the withdrawal of progesterone is the initiator of parturition, and they have demonstrated the importance of the concentrations of PRs A and B and their relationship in the mechanism of parturition [5–10, 12]. Currently, other aspects are also known to be required for the onset of parturition. Cervical changes, for example, are associated with the expression of receptors that modify local mechanisms such as the remodelling of collagen, the migration of inflammatory cells, and changes in the extracellular matrix as a whole [9, 13–15].

To quantify the concentrations of ORs and PRs in the myometrium during labour, a prospective study including 21 patients was designed to evaluate the concentrations of these receptors on the surfaces of myometrial cells in tissue biopsies collected during a caesarean section of full-term pregnant women in relation to the presence or absence of labour.

## 2. Materials and Methods

Myometrial tissue was obtained by biopsies performed during caesarean sections of 21 full-term pregnant women (37 to 41 weeks). Six women were in labour (mean 39 weeks, range 37 to 41), and 15 were not in labour (mean 39 weeks, range 37 to 41 weeks). All subjects signed the informed consent form prior to their inclusion in the study. The study was evaluated and approved by the Research Ethics Committee of the Federal University of São Paulo (UNIFESP). Gestational age was confirmed by the date of the last menstrual period and/or by ultrasonography in the first and/or second trimesters. Women were considered parturient based on the assessment of the signs and/or symptoms of labour: a Bishop score greater than or equal to 5, two or more uterine contractions lasting more than 40 seconds in 10 minutes, cervical dilation of 2 cm in primiparous women and 3 cm in multiparous women, and insinuated foetal presentation.

The indications of cesarean section in patients during the labour were as follows: two or more previous cesarean sections, fetal distress observed during the evolution of the labour, and primiparous women in breech. The exclusion criteria were patients with obstructed labour, clinical, and/or obstetrical complications, and patients undergoing induction/conduction of labour with oxytocin or prostaglandin, using medication except multivitamins.

During the caesarean section (after placental expulsion), a small piece of the myometrium was removed at the upper middle portion of the incision (hysterotomy) by the researcher (JSA), always using the same technique: a resection of 0.5 cm³ of the myometrium with the aid of a scalpel, excluding the endometrium and visceral peritoneum, and then proceeding to a normal suture of the hysterectomy. The material obtained from each biopsy was placed in 10% buffered formaldehyde for a maximum of 48 hours and subsequently processed in an Autotechnicon (Autotechnicon 2^A^, Chauncey, NY, USA) for 12 hours (overnight), using alcohol, xylene, and paraffin. After processing, the specimens were placed in cassettes and embedded in paraffin (Soldan Cytological Products Ltd., Porto Alegre, RS, Brazil). The paraffin blocks containing the histological processed materials were cooled in a freezer and cut on a rotary microtome to obtain 4-micron-thick histological sections using disposable razors (Leica) suitable for this purpose. The paraffin ribbons obtained from the microtome sectioning process were carefully placed in a histological water bath, and the sections were deposited on precleaned slides coated with a special adhesive that improves tissue adhesion (VER-SA PLUS, Euriegas). The slides were then placed in a laboratory oven for 1 hour. 

The immunohistochemical preparation procedures were as follows. (1) Preparation of the histological sections: washing in xylene at room temperature for 15 minutes, twice, followed by 100% ethanol (3 times) for 30 seconds each, 95% ethanol for 30 seconds, 80% ethanol for 30 seconds, and 70% ethanol for 30 seconds, with a final washing in running and distilled water. (2) Antigen retrieval: the slides were incubated in moist heat and incubated in citrate buffer (10 nM, pH 6.0) in a steamer (T-Fal) for 20 minutes.

The immunohistochemical visualisation consisted of the following steps in a reaction using the streptavidin-biotin peroxidase complex (StreptABC). (1) The slides were incubated with a specific antibody (monoclonal ER (1B)/PR (2B)) for 30 minutes in a moist chamber. (2) The slides were washed in Tris-buffered saline (Dako TBS), and the buffer was exchanged every 3 minutes. (3) The slides were then incubated with biotinylated secondary antibody (anti-immunoglobulin from the animal species from which the specific antibody was obtained) in a moist chamber for 30 minutes at 37°C. (4) The slides were again washed in buffer with three exchanges, once every 3 minutes. (5) Revelation and mounting: the slides were incubated in a substrate solution (diaminobenzidine; DAB, 60 mg%) for 3 minutes at 37°C in the dark. The positive control slides were then observed through the microscope for the development of a golden-brown precipitate, the final product of the reaction; the slides were washed in running and distilled water for 3 minutes, counterstained with Harris haematoxylin for 30 seconds, and washed in running and distilled water. The slides were then dehydrated in 50% ethanol, 80% ethanol, 95% ethanol, and 100% ethanol (3X) followed by xylene (3X), mounted in Entellan cover slips (Merck 1.07961) and examined under an optical microscope by three pathologists to confirm the suitability of the tissue sample. The pathologists also verified whether scarring from previous caesarean sections would interfere with the results, which was avoided by observing specific areas for study. 

The quantitative analysis of the presence of ORs and PRs was performed using a computerised image-analysis program (ImageLab). For the quantification of the areas occupied by the two hormone receptors, microscopic fields of 422 *μ*m × 322 *μ*m were captured at a magnification of 100x while maintaining several standardised settings of the optical microscope (Leitz Carbelux Model 6MBH). A Leitz Wetzlar-160/0.17 Plan 10/0.45 lens was used at maximum light intensity with the maximum condenser aperture at a distance of 6.5 cm from the slide.

The digitalised fields had a horizontal resolution of 640 pixels and a vertical resolution of 480 pixels in 24-bit colour (16 million colours), and the same range of colours for the area to be quantified was used in each of the fields. The colour range was adjusted for isolation in areas that represented the ORs and PRs in the image. The averaged values obtained from both analysed fields in each slide were determined by considering two parameters: the percentage of the area occupied by hormone receptors and the optical density of the light reflected by each of the receptors. To avoid possible subjectivity, all examined materials were identified by each patient's medical record number, and the collected data and the results obtained were compared only at the end of the study. The pathological anatomy laboratory technician numbered the patients according to the order of entry of the biopsy material in the laboratory, enabling the statistical analysis of patients numbered 1 to 21. The name and number of the medical record were retained, allowing for easy identification. Therefore, the patients in labour and not in labour could readily be compared at the end of the study following the examination of the slides.

All data were stored in Excel 2007 (Microsoft Corp., Redmond, WA, USA) spreadsheet and analyzed by the Statistical Package for the Social Sciences (SPSS) program, version 19.0 for Windows (SPSS Inc., Chicago, IL, USA). The Mann-Whitney test was used to compare the pregnant women in each study group with respect to the numbers of ORs and PRs. The Wilcoxon test was used to compare the numbers of ORs with the number of PRs in each group. Spearman's correlation coefficients (*r*) were used to study the possible relationships between ORs and PRs in each group separately. The level of significance (*P*) was <0.05. The significant values were marked with asterisks. 

## 3. Results

The medians of PRs and ORs in pregnant women in labour (Group I) were 29.3 ± 2.38 (range, 24.6–30.2) and 32.3 ± 10.92 (range, 22.9–49.0), respectively. The medians of PRs and ORs in pregnant women without labour (Group II) were 43.6 ± 14.94 (range, 23.6–70), and 43.9 ± 13.17 (range, 18.3–62.6), respectively. We did not observe significant differences of the number of ORs and PRs in both groups by the Mann-Whitney test (*P* = 0.13 and 0.37, resp.). 

The *Z* critical between both groups was 1.96 (oestrogen-*Z* calculated = 1.56 and progesterone-*Z* calculated = 0.93), proving difference in the concentration of both receptors in the myometrium of lower uterine segment of pregnant women during and without labour (Table 1).

The number of ORs was statistically more than that of PRs in the myometrium of lower uterine segment of Group II (Group I *Z* calculated = 0.94 versus Group II *Z* calculated = 16.00). We observed that the PRs and ORs concentrations were smaller in the myometrium of lower uterine segment of Group I, and these concentrations were higher in Group II (Group I *r* calculated = 0.49 versus Group II *r* calculated = 0.74) (Table 2). 

## 4. Discussion

Knowledge of the mechanism of parturition should allow patients in preterm labour and patients with postterm pregnancy to be properly treated, minimising the complications of such conditions [2, 3, 14, 16]. Recent works have suggested that a decrease in the number of progesterone B receptors, favouring an increase in the expression of progesterone receptor A, results in a triggering of the mechanism that initiates labour [5–7, 9, 10]. Thus, these authors suggest a functional withdrawal of progesterone.

The results of the present study suggest that the downregulation of the concentration of PRs may be associated with human parturition, or at least with its initiation. Because no significant changes were noted in ORs numbers in either labour or nonlabour conditions, oestrogen action may be held constant. A constant level of oestrogen may also maintain the status of both ORs and PRs, given the well-known upregulation of both hormone receptors by oestrogen and the downregulation of these receptors by progesterone [1, 2, 4, 11]. Another implication of the sustained OR levels may be explained by the observation that oestrogen excites the myometrium, increases the levels of oxytocin receptors, and induces the inflammatory process currently known to be involved in parturition [5, 6, 9].

The present study confirmed the findings of several previous studies, particularly from the study that served as our model, that is, the upregulation of ORs and PRs. When PR numbers decrease, there is a less expressive decrease in OR numbers, and PRs apparently tend to decrease in patients in labour [8, 10]. We note that the present study was conducted in the myometrium of lower uterine segment and, similarly to the studies conducted by Rezapour et al. [8] and Winkler et al. [10], we observed a low concentration of PRs in the myometrium of lower uterine segment. The study by Merlino et al. [6] required a correction of the receptor analysis due to the small number of myometrial cells in the uterine cervix, which is also consistent with findings of the present study.

Similarly to the study of Winkler et al. [10], in the present study, a tendency for a decrease in the number of PRs was observed in pregnant women at the beginning of labour with a Bishop score equal to or less than 5. According to study [6], the fact that lower concentrations of PRs were found may have been due to a missing analysis of a specific receptor type. The authors showed that the ratio of PR-A to PR-B increased during labour. They also studied pregnant women subjected to elective caesareans, in both term and preterm pregnancies, although not in labour. Their results showed that a PR-A/PR-B ratio of 0.5 was maintained in the term and preterm pregnancies, whereas during labour, the ratio doubled in favour of PR-A, with a value of 2.5/1. They concluded that “these findings are consistent with previous studies and support the hypothesis that human parturition involves a specific increase in the ratio of PR-A/PR-B in the myometrium due to an increased expression of PR-A” [6]. This result drew special attention to the difference in hormone concentrations in pregnant women who had no signs or symptoms of labour. Significant differences were found between the concentrations of hormone receptors in nonparturient women, whereas no difference was noted in parturient women; that is, there appeared to exist a constancy in the numbers of steroid receptors when the myometrium was clearly in labour activity, which may be explained by the postulate of Mendelson stated above [5].

The differences in the methodologies used to assess the concentrations of the studied hormone receptors in our study and others allowed for a corroboration of the results obtained in other analyses using different methods. Thus, it is worth noting that the study and quantification of OR and PR is less laborious and costly using the methodology developed in the present study. Furthermore, this methodology can be widely applied in other researches. The similarities between this study [6] and the present study are the following: the patients were subjected to undergo elective caesarean sections; that is, they were not in labour; parturients were subjected to caesarean section due to obstetric requirements and distributed according to clinical criteria for the assessment of the presence or absence of labour; biopsies were obtained from the myometrium of lower uterine segment, at the upper portion of the hysterotomy incision; and we attempted to remove the whole tissue that was not myometrium. However, the authors note that, despite the rigour adopted for myometrial tissue removal, there may have been some contamination with other tissues.

In an other study on the myometrium, Merlino et al. [7] investigated the expression of the nuclear progesterone receptor in foetal membranes and decidua at term, before and after the onset of labour. Their results suggested that the decidua, rather than the foetal membranes, is the direct target for the nuclear PR during pregnancy because the receptor was only detected in the decidua. 

The limitations of this study were the small sample. Our hard criteria to include only pregnant women with spontaneous labour (Group I) and to exclude the dystocia as indication of caesarean section contributed to this small sample. 

The present study is in accordance with the literature, showing the changes in steroid receptor sites and assisting in understanding the possible mechanism of labour and how they can intervene in this situation if necessary, for example, in preterm and prolonged gestation. Finally, the need for an animal model with a parturition physiology similar to the human system is also required, allowing more detailed studies to be performed *in vivo*.

## Figures and Tables

**Table 1a:** (a)

	Oestrogen		Progesterone	
	I	II	I	II
	39.9	53.3	29.8	60.0
	39.3	55.4	30.2	48.3
	49.0	62.7	28.9	53.8
	23.4	56.6	29.7	62.6
	22.9	70.0	24.6	50.4
	25.3	23.6	25.7	22.1
		44.1		30.4
		37.5		33.9
		43.6		20.4
		44.0		25.1
		30.0		29.5
		36.3		37.9
		34.2		30.1
		32.8		22.5
		34.4		18.3

Mean	33.3	43.9	28.2	36.4
Median	32.3	43.6	29.3	30.4

**Table 1b:** (b)

Mann-Whitney test	
(Group I versus Group II)	
*Z* critical = 1.96	
Oestrogen	Progesterone

*Z* calculated = 1.56	*Z* calculated = 0.93

**Table 2a:** (a)

	Group I		Group II	
	Oestrogen	Progesterone	Oestrogen	Progesterone
	39.9	29.8	53.3	60.0
	39.3	30.2	55.4	48.3
	49.0	28.9	62.7	53.8
	23.4	29.7	56.6	62.6
	22.9	24.6	70.0	50.4
	25.3	25.7	23.6	22.1
			44.1	30.4
			37.5	33.9
			43.6	20.4
			44.0	25.1
			30.0	29.5
			36.3	37.9
			34.2	30.1
			32.8	22.5
			34.4	18.3

Mean	33.3	28.2	43.9	36.4
Median	32.3	29.3	43.6	30.4

**Table 2b:** (b)

Spearman's correlation coefficient	
(Oestrogen versus progesterone)	
Group I	Group II

*r* calculated = 0.49	*r* calculated = 0.74*
*r* ^2^ = 0.24	*r* ^2^ = 0.55

**Table 2c:** (c)

Wilcoxon test	
(Oestrogen versus progesterone)	
*Z* critical = 1.96	
Group I	Group II

*Z* calculated = 0.94	*Z* calculated = 16.00*
	Oestrogen > progesterone
